# ASSESSEMENT OF BONE AGE AGREEMENT BETWEEN THE SAUVEGRAIN AND GREULICH AND PYLE METHODS

**DOI:** 10.1590/1413-785220243204e278912

**Published:** 2024-10-07

**Authors:** Beatriz Nogueira Leite, João Vitor Nogueira Rubez, Carlos Alberto Arruda Soufen, Bruna Zanetti Pereira, Marcos Vinicius Felix Santana, Eiffel Tsuyoshi Dobashi

**Affiliations:** 1. Hospital IFOR – Rede D’or, São Bernardo do Campo, SP, Brazil; 2. Brazilian Society of Orthopedics and Traumatology (SBOT), São Paulo, SP, Brazil; 3. Universidade Federal de Sao Paulo Unifesp, Faculdade de Medicina, Departamento de Ortopedia e Traumatologia, Sao Paulo, SP, Brazil

**Keywords:** Child, Puberty, Radiography, Evaluation Study, Age Determination by Skeleton, Observer Variation, Criança, Puberdade, Radiografia, Estudo de Avaliação, Determinação da Idade pelo Esqueleto, Variações Dependentes do Observador

## Abstract

**Objective:**

To evaluate the intra and inter observer agreement of the Sauvegrain, Greulich and Pyle methods.

**Material and methods:**

This is an observational, retrospective and cross-sectional study ethically approved by opinion 6,192,391. 100 radiographic images of the elbow and 100 of the left wrist and hand were collected from children whose images were selected by a researcher who did not carry out the evaluations. The Sauvegrain, Greulich and Pyle methods were used to determine bone age. We provided a detailed explanation of each method and the evaluators received a file with the study images. After three weeks, the exams were randomized and the radiograms were reevaluated. Of the 100 patients in group A, 61 (61%) were boys and 39 (39%) were girls. In group B, 67 (67%) were boys and 33 (33%) were girls.

**Four statistical analyzes were used:**

correlation; intraclass correlation; analysis using the Bland-Altman graph; differences between groups.

**Results:**

Intra and interobserver agreement between groups was considered excellent.

**Conclusions:**

Despite the excellent agreement, group A presented a significantly better value than B. Biological ages show a greater difference compared to chronological ages in group A. In group B, skeletal and chronological ages do not show statistical difference according to the accuracy test. ***Level of Evidence III, Cross-Sectional Observational Study.***

## RESUMO

**Objetivo:**

*Avaliar a concordância intra e interobservadores dos métodos de Sauvegrain e Greulich e Pyle.*

**Material e métodos:**

*Trata-se de um estudo observacional, retrospectivo e transversal, aprovado eticamente pelo parecer 6.192.391. Foram coletadas cem imagens radiográficas do cotovelo e cem do punho e mão esquerdos de crianças, selecionadas por um pesquisador que não realizou as avaliações. Utilizou-se os métodos de Sauvegrain e Greulich e Pyle para determinar a idade óssea. Uma explicação detalhada de cada método foi realizada, e os avaliadores receberam um arquivo com as imagens do estudo. Após três semanas, os exames foram randomizados e os radiogramas reavaliados. Dos cem pacientes do grupo A, 61(61%) eram meninos e 39(39%) meninas. No grupo B, 67(67%) eram meninos e 33(33%) meninas.*

**Quatro análises estatísticas foram utilizadas:**

*correlação; correlação intraclasse; análise pelo gráfico de Bland-Altman; e diferenças entre grupos.*

**Resultados:**

*A concordância intra e interobservador entre os grupos foi considerada excelente.*

**Conclusões:**

*Apesar da concordância excelente, o grupo A apresentou valor significantemente melhor que o B. As idades biológicas apresentam maior diferença frente as idades cronológicas no grupo A. No grupo B, as idades esqueléticas e cronológicas não apresentam diferença estatística segundo o teste de acurácia.* ***Level of Evidence III, Cross-Sectional Observational Study*** .

## INTRODUCTION

The practical application of determining skeletal age is widely used in pediatric orthopedics, forensic medicine, and pediatric endocrinology. Correcting length discrepancies between limbs, deformities, and scoliosis, among other things, requires appropriate knowledge to make an assertive decision about the moment and appropriate intervention, conservative or operative.

Any growing skeletal structure can be used to assess biological age. When researching the medical literature, we observed that, over the years, different methods were developed for the study and clinical application of this variable, such as that of Oxford (1957), Risser (1958), Sauvegrain et al. (1962), and Greulich and Pyle (1950). Despite the typical application of these systems, there is no definition of which presents a greater degree of trust and agreement among those who use this knowledge. The scarcity of scientific works addressing this topic was decisive for this study.

Bone age analysis determines developmental bone growth and maturation in ordered sequences. Any region of the skeleton that has growth is known to be usable in the pediatric population. To properly carry out these assessments, x-rays can be used and must be obtained using an appropriate technique. Such care aims to avoid and resolve errors in determining skeletal age. In this regard, poor positioning of the studied segment is considered the most common error.

 Among the different systems, we have the Risser system, which uses the ossification of the iliac process and presents five stages that represent the evolution of the fusion of this structure. The interpretation of this parameter dramatically helps in choosing the appropriate treatment for scoliosis. The lower the Risser stage, the greater the patient’s expected remaining growth. It is considered easy to apply and is interpreted using radiography in the anteroposterior view of the spine. On x-rays of the pelvis, other ossification centers can be visualized, such as that of the triradiate cartilage. This is directly related to the peak velocity during growth ^1^ . 

The Greulich and Pyle method uses radiographs of the bones of the hand and wrist on the left side to study the ossification centers of each anatomical structure in this segment. After this investigation, a score defines the degree of skeletal maturity where the result is correlated with the chronological age of the patient involved.

 The systematics of Sauvegrain et al. uses radiographs of the elbow in anteroposterior and lateral views and is more effective when applied to pediatric patients in the first two years after the onset of puberty ^2^ . The pubertal period is characterized by an increase in growth speed and the emergence of secondary sexual characteristics as stipulated by Tanner’s criteria. In girls between nine and 13 years old and boys between 11 and 15, the composition of the elbow is still predominantly cartilaginous. Therefore, any radiographic change is naturally recognized at this age, making supporters of this method consider it more reliable than Greulich and Pyle. In 2005, Dimeglio et al. added three intermediate scores: 3.5 for the trochlea, 6.5 for the olecranon, and 5.5 for the proximal radial epiphysis. According to the authors, this update increased the degree of reliability of this method ^3^ . Furthermore, scores in boys and girls have been documented as directly related to growth speed ^1^ . 

 Naik et al. ^4^ demonstrated that Sauvegrain et al.’s method is highly reproducible and allows for agreement between the assessments of three observers. 

Given the above, the authors of this study aim to evaluate intra and interobserver agreement using the methods of Sauvegrain et al. and Greulich and Pyle for determining bone age.

## MATERIAL AND METHODS

This is an observational, retrospective, and cross-sectional study. The project for this research was submitted for ethical consideration and approved for conduction under CAAE opinion 6,192,391.

Two groups were formed, and 100 radiographic images of the elbow and 100 radiograms of children’s left wrist and hand were collected from our service’s radiographic image storage bank.

These were selected by a research member who did not participate in the radiographic examination classification process. The adequate quality of the exams, strictly following the inclusion and non-inclusion criteria determined by the study authors, was decisive in choosing the radiographs.

### Inclusion criteria

- Participants between six and 16 years of age;
- Both sexes;
- Patients with a history of trauma to the left elbow, left wrist, and left hand who were assessed for suspected fracture of the upper limb but without evidence of bone injury;
- Patients with elbow radiographs in anteroposterior and lateral views of good technical quality;
- Patients with radiographs of the left wrist and left hand in the anteroposterior view of good technical quality;
- No history of previous fracture, congenital or acquired anatomical changes;
- Signature of the Informed Consent Form (ICF) by parents or guardians.

### Exclusion criteria

- Not meeting the inclusion criteria.
- Not signing the TCLE.

Three different researchers applied Sauvegrain et al.’s and Greulich and Pyle’s methods: the former analyzed elbow radiographs (group A), and the latter determined bone age by studying radiographic examinations of the left hand and wrist (group B).

**Figure 1.**
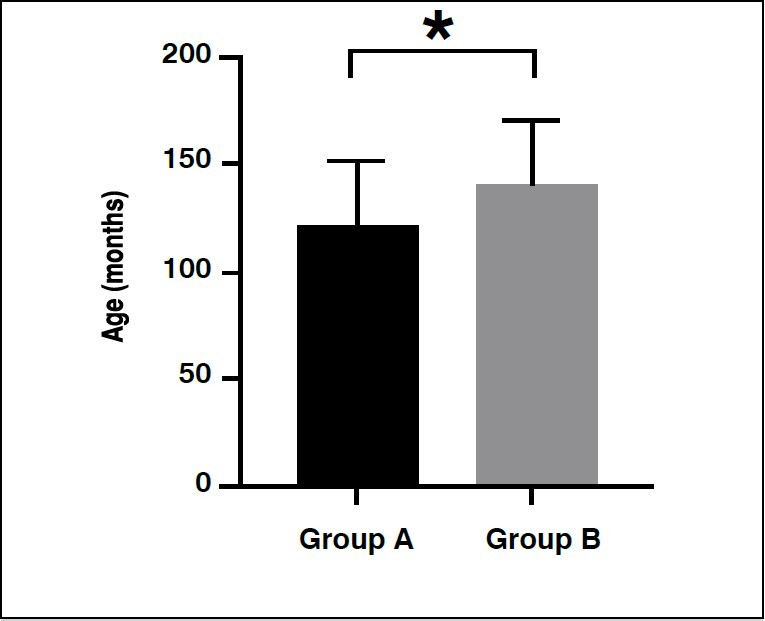
Means and standard deviations of the ages of 200 patients according to groups

 Two hundred patients were studied, 100 from group A and 100 from group B. Of the 100 patients in group A, 61(61%) were male and 39 (39%) were female. In group B, 67 (67%) were male and 33 (33%) were female. In terms of age, group A was significantly younger than B, with ages ranging from 73 to 190 months (6 to 15 years), whereas patients in group B were between 73 and 195 months (6 to 16 years) ( Table 1 and Figure 1 ). 

**Table 1.** Mean and standard deviation values or absolute frequencies of ages and sexes of the 200 patients assessed

Variable		Group A (n = 100)	Group B (n = 100)	p
Sex	Male	61	67	0.462
	Female	39	33	
Age (months)		122.5 ± 30.3	138.7 ± 32.7	< 0.001

* p < 0.05

A detailed meeting with explanations of each system used in this work was held to minimize interpretation bias. A file with two groups of images was made available to evaluators. Each of the researchers independently and confidentially classified the radiograms. They were instructed not to discuss the results until the study was completed to avoid an erroneous correlation increase.

Each observer had the classifications as a reference, with drawings of all of them and the time needed for them to evaluate the radiographs.

After three weeks, the same material was randomized and subjected to a second evaluation following the previously described method.

 A professional in the field of Medical Statistics carried out the statistical analysis of the results obtained. Four approaches were developed to evaluate precision and accuracy in the study: i) correlation analysis, ii) intraclass correlation (ICC) analysis, iii) analysis using the Bland-Altman graph, and IV) analysis of differences between groups. Each of these analyses complements the information of the others. In contrast, the simple correlation analysis shows the association between two measurements (the two readings of the same image by the same evaluator or the relationship between the measurement and chronological age). Intraclass correlation analysis (which can be used in intra and intraclass analyses) compares the results of two or more evaluators. It considers the general relationship and the specific agreement between the observed numerical values ^5^ . The Bland-Altman plot presents the relationship between the measurement size and the numerical difference between the results of the methods. This can help to understand whether there is an error pattern between the measurements. Finally, analyses of group differences present the probability that the compared values differ. 

Spearman’s correlation was used for correlation analyses since the data did not present a normal distribution according to the Shapiro-Wilk test. For the intraclass analysis, we chose to use the two-way mixed-effect model with the option of an absolute agreement relationship (since there is an interest in obtaining equal numerical values between the two measurement methods or between the evaluators). Differences between groups were evaluated using non-parametric Wilcoxon and Friedman tests, depending on the number of groups evaluated. In these assessments, the absolute values of estimated ages or obtained points were compared between different observers or times, and the difference between observations was assessed if it was significantly different from zero.

Ages between groups were compared using the Mann-Whitney test, and the sex ratio using Fisher’s exact test.

The comparison between the correlation coefficients and ICC used the method proposed by Eid et al. (2010).

Diagnostic randomization systems were used to analyze general patterns and accuracy, to not smooth out errors by using mean values.

 The scoring data were transformed into chronological age in months, as recommended by the method of Sauvegrain et al. ^2^ to evaluate the accuracy of group A. 

Statistical analyses and graphs were developed using the R program, considering a significance level of 5% (α = 0.05).g

## RESULTS

### Intraobserver assessment

The data demonstrate that the methods used present excellent internal agreement for groups A and B. This can be observed by:

- Lack of significant differences between the absolute values at the different time points evaluated.
- High significant correlations between.
- Lack of significant difference between the measurement differences and the zero value
- Significant high ICC values.

This pattern of results was constant for both group A ( Table 2 ) and B ( Table 3 ). Importantly, all correlation values were positive and above 0.75, which characterizes them as directly proportional and robust. For ICC, it is possible to use the interpretation proposed by Koo and Li (2016): values below 0, 5 show low agreement, between 0.5 and 0.75, moderate agreement, from 0.75 to 0.9, good agreement, and above 0.9, excellent agreement. 

 When comparing the ICC correlation coefficients between the groups, it was possible to observe a significant statistical difference with higher values for group A ( Table 4 ). 

**Table 2.** Significance, correlation, and ICC values of 100 patients assessed according to group A by three evaluators at two different time points

Evaluator	Absolute values*	Correlation between time points		Difference from 0**	ICC	
	p	r	p	p	ICC	p
1	0.984	0.98	< 0.001	0.901	0.986	< 0.001
2	0.829	0.98	< 0.001	0.321	0.983	< 0.001
3	0.821	0.98	< 0.001	0.245	0.988	< 0.001
General	0.900	0.96	< 0.001	0.869	0.991	< 0.001

* Comparisons between the absolute values of the two time points evaluated

** Comparison between the differences concerning the two time points and the zero value

**Table 3.** Significance, correlation, and ICC values of 100 patients assessed according to group B by three evaluators at two different time points

Evaluator	Absolute values*	Correlation between time points		Difference from 0**	ICC	
	p	r	p	p	ICC	p
1	0.623	0.97	< 0.001	0.623	0.968	< 0.001
2	0.279	0.89	< 0.001	0.279	0.913	< 0.001
3	0.424	0.96	< 0.001	0.424	0.979	< 0.001
General	0.408	0.98	< 0.001	0.388	0.982	< 0.001

* Comparisons between the absolute values of the two time points evaluated

** Comparison between the differences concerning the two time points and the zero value

**Table 4.** Correlation coefficient and intraclass correlation in 200 patients assessed according to groups

Measure	Group A	Group B	p
Correlation	0.964	0.978	0.061
ICC	0.991	0.982	0.008

## Interobserver assessment

 The analyses of interclass differences showed that, in both groups, the agreement between the evaluators was relatively high, with no significant difference between the coefficients ( Table 5 ). 

**Table 5.** Interclass correlation coefficient values in two different groups in 200 patients assessed

Group	ICC	p
A	0.985	< 0.001
B	0.982	< 0.001
p *	0.261	

* p-value associated with the comparison between ICC of different anatomical locations

 The accuracy assessment showed a moderate relationship between chronological age and the estimates generated by the assessment of group A ( Table 6 ), with values even significantly different between the estimates and absolute values. 

**Table 6.** Significance, correlation, and ICC values of 100 patients assessed according to estimates and chronological ages for group A

Evaluator	Absolute values*	Correlation between estimate and actual value		Difference from 0**		ICC
	p	r	p	p	ICC	p
1	< 0.001	0.88	< 0.001	< 0.001	0.721	0.004
2	< 0.001	0.89	< 0.001	< 0.001	0.678	0.017
3	< 0.001	0.83	< 0.001	< 0.001	0.648	0.007
General	< 0.001	0.87	< 0.001	< 0.001	0.669	0.010

* Comparisons between absolute values of chronological age and estimate

** Comparison between the differences concerning the two measurements (estimate and chronological) and the zero value

**Figure 2.**
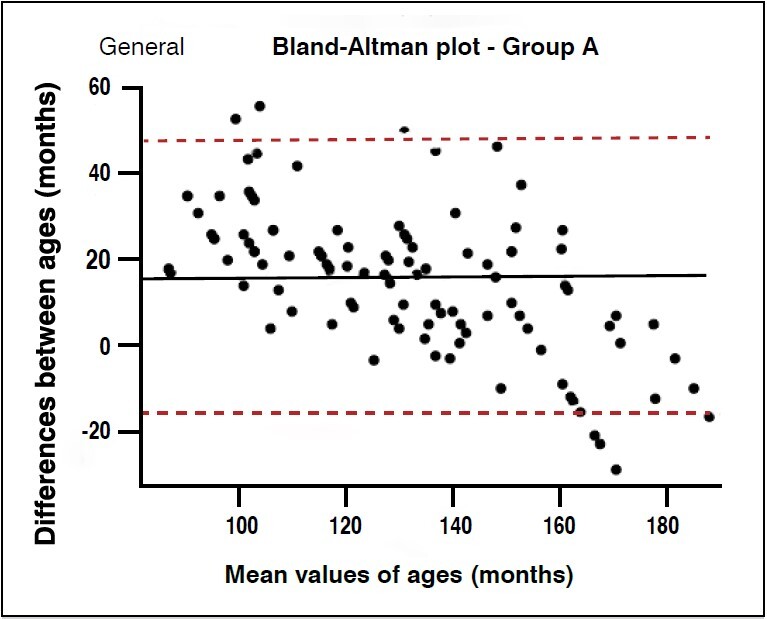
Bland-Altman plot for chronological age accuracy in group A assessments in 100 patients.

**Figure 3.**
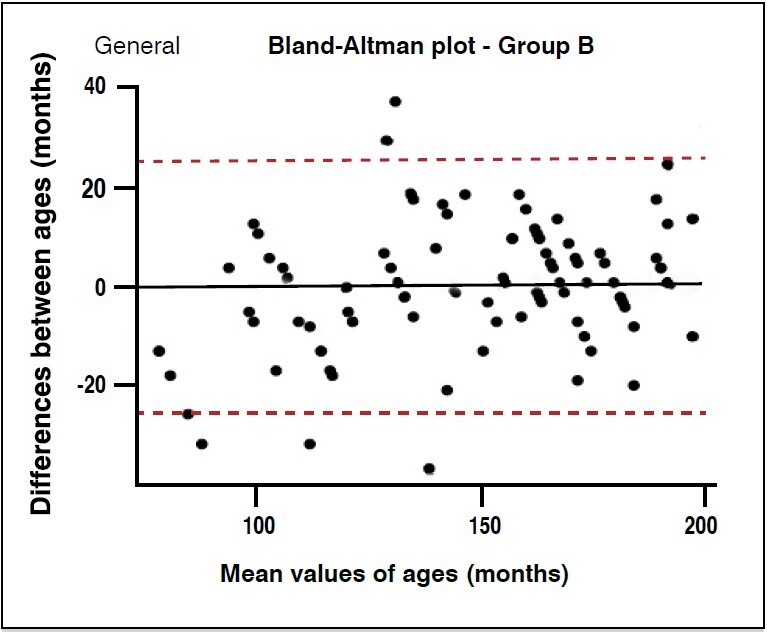
Bland-Altman plot for chronological age accuracy in group B assessments in 100 patients.

 The Bland-Altman plot for accuracy analysis showed that for group A, the values were far from zero, with a large part of the estimates above the chronological value. This pattern was constant across all evaluators and overall assessment ( Figure 2 ). For group B, it was possible to demonstrate a balanced distribution of ages above and below the chronological variable, with few values outside the confidence interval ( Figure 3 ). 

 Accuracy concerning group B showed results with strong, simple correlations and excellent ICC. However, in one of the evaluators, there was a significant discrepancy between the differences in estimates and the value of chronological age, reducing the confidence in this result ( Table 7 ). 

 There was a significant difference in accuracy between groups, with higher values for group B ( Table 8 ). 

While both groups presented excellent results in the intraobserver agreement, group A (elbow) presented a significantly better value than group B (wrist), possibly due to using a point scale. In this case, there is variation, and different ages receive the same value.

Regarding interobserver agreement, both groups presented excellent values without significant differences.

 Accuracy was moderate for group A and excellent for group B, with significant differences ( Table 9 ). 

**Table 7.** Significance, correlation, and ICC values of 100 patients assessed according to estimates and chronological ages for group B

Evaluator	Absolute values*	Correlation between time points		Difference from 0**	ICC	
	p	r	p	p	ICCc	p
1	0.969	0.94	< 0.001	0.815	0.929	< 0.001
2	0.362	0.89	< 0.001	0.046	0.875	< 0.001
3	0.701	0.93	< 0.001	0.745	0.922	< 0.001
General	0.556	0.94	< 0.001	0.181	0.981	< 0.001

* Comparisons between absolute values of chronological age and estimate

** Comparison between the differences concerning the two measurements (estimate and chronological) and the zero value

**Table 8.** Accuracy values for two anatomical locations in 200 patients assessed

Measure	Group A	Group B		P
ICC	0.669	0.982		< 0.001

**Table 9.** Intra and interobserver agreement values of 100 patients for group A and group B, respectively

Intraobserver agreement	Group A	Group B	p
Evaluator 1	98.6%	96.8%	0.002
Evaluator 2	98.3%	91.3%	< 0.001
Evaluator 3	98.8%	97.9%	0.009
General	99.1%	98.2%	0.008
Interobserver agreement	Group A	Group B	p
General	98.5%	98.2%	0.261

## DISCUSSION

Skeletal maturity can be assessed in several ways, but the most frequently used methods in medical practice are Greulich and Pyle and Tanner-Whitehouse II. The latter has a higher degree of reproducibility but is time-consuming and considered difficult to apply.

 Roche et al., Acheson et al., and Milner et al. found that the bone ages estimated by the GP method were lower than those by the Tanner method. Waldmann et al., Roche et al., and Fry found opposite results: the values obtained by the Tanner method were higher concerning chronological age in both sexes, being higher for females ^6^^-^^10^ . 

We found a study that evaluated 114 normal individuals aged 2 to 21 years, analyzed five methods: cervical vertebra (Hassel-Farman), iliac crest (Risser), hip (Oxford), knee (O’Connor), calcaneus (Nicholson) and applied to EOS. The intra and interobserver agreements were excellent, except concerning the knee method (0.865 – good). The calcaneal and cervical exams were the quickest to perform (average of 17,5 s, 33,4 s per evaluation). While the authors concluded that bone age assessment is possible with all five methods, the method proposed by Hassel-Farman proved to be easier, faster, and more reliable.

The simplicity, convenience, and speed make the Greulich and Pyle method the most commonly used reference standard for assessing skeletal age. This is widely used despite requiring a manual process that is more time-consuming than many other simple radiographic examinations. An atlas in electronic format could be developed to integrate into everyday work.

Critics of the method report a high variability of results to the detriment of defenders who point to greater reproducibility.

Knowing or not knowing chronological age before evaluating bone age radiographs does not differentially affect inter and intraobserver reliability. However, observers will likely interpret the radiograph as normal when chronological age is known.

 Alternative atlases – Skeletal Development of the Hand and Wrist: Digital Bone Age Companion (DBAC) (Oxford University Press, New York) – have been developed for skeletal age estimation. However, no work has compared its applicability and comparison to the Greulich and Pyle ^11^ . 

DBAC is a commercially available application that accompanies a bone age reference book. Precursor images have been digitally edited so that the developmental characteristics of each bone in the left hand and wrist match Greulich and Pyle’s standards.

 Some authors report a potential bias when employing automated context integration by age and sex using DBAC. For example, Berst et al. ^12^ observed that evaluators are more likely to interpret radiography with normal results when chronological age is known. 

 A particular applicability of knowledge regarding bone age is in Legg-Calvé-Perthes disease, in which there is a delay in the skeletal growth of children with it and some cases of arrested skeletal development. Loder et al. document a delay in pelvic and hand bone age in children with this disease. However, for girls, the bone age of the pelvis was similar to the bone age of the hand-wrist ^13^ . Burwell et al. state that Legg-Calvé-Perthes disease is an acromelic disorder concerning anthropometric measurements ^14^ . The forearm and hand present more significant growth impairment than the arm, which also occurs between the foot and the tibia. 

Since Risser’s study in 1958, it has been widely used. More than 20 years ago, Goldberg et al. found acceptable interobserver reliability for the Risser stage (Kappa = 0.8). This author’s stages are reliable radiographic parameters to assess the growth potential in children with scoliosis. In addition to acceptable interobserver agreement and clinical utility, iliac apophysis stages reflect local biology. The histological stage of iliac apophyseal chondrocytes was also inversely correlated with the Risser stages. This is a helpful indicator of growth potential in adolescent idiopathic scoliosis. The Risser should be used with other tools, such as skeletal age, chronological age, and menarche in girls.

A weakness of the Risser staging system is related to greater progression of the scoliotic curve with greater speed in height gain. While Risser Stage 4 has commonly been considered a point at which curve progression stops, the interruption of curve progression continues until Stage 5.

 To improve the correlation of curve progression and the Risser staging system derived from the Sauvegrain method, Demeglio et al. proposed a simplified system that evaluates the morphological development of the olecranon to determine the growth acceleration phase ^2^^,^^3^ . The simplified olecranon method was validated and proposed for patients with scoliosis to determine skeletal age and peak growth velocity, making Risser stage zero more useful. 

Based on our results, Sauvegrain et al.’s method was considered advantageous compared with Greulich and Pyle’s.

 According to some authors, Sauvegrain et al.’s method is dynamic because the morphological changes apparent on elbow radiographs are straightforward to assess ^2^ . At the beginning of puberty, the elbow still has a large amount of cartilaginous content, and after two years, the fusion of the ossification centers will be complete. The Greulich and Pyle system does not consider this critical period. This practical method allows radiographic interpretation in less than a minute and is also highly reproducible. 

However, this method has limitations, as it is restricted to the period of the pubertal growth spurt and the year before this phase.

The method offers the possibility of dividing puberty into two phases: acceleration and deceleration. The acceleration in growth velocity or upward phase of pubertal growth occurs between 11 and 13 years of skeletal age in girls and between 13 and 15 years in boys. The growth centers of the elbow are open and progressively ossify during this phase. The deceleration in the growth rate or downward phase of pubertal growth occurs between 13 and 16 years of skeletal age in girls and between 15 and 18 years in boys.

In an imaging exam, the individual analysis carried out by humans is expected to add a bias in quantifying and predicting the outcomes based on its assessment. Indeed, a growing number of studies have discussed computerized analysis methods and artificial intelligence in medical practice.

Our study was conducted with three evaluators with the same level of training and qualifications. Evaluators with different levels of training, a longer time interval for carrying out the analyses, and a more significant number of cases evaluated should not modify the results of the analyses when the classification system is appropriate. This premise supports the idea that an ideal classification system must comply with a series of well-defined criteria, such as being easy to apply, highly reproducible, indicating the appropriate treatment to use, and providing us with a prognosis. It should also allow comparisons between the results obtained from different series to be compared. This fact is observed in our study, which demonstrated excellent inter-rater reliability.

As a limitation of the study, we point out that the sample analyzed did not reach the number of participants determined by the sample calculation.

## CONCLUSIONS

While both groups presented excellent results in intraobserver agreement, group A presented a significantly better value than group B, possibly due to using a point scale. In this case, there is the same variation, and different ages receive the same value.

Interobserver agreement in both groups showed excellent values without significant differences.

In individuals in group A, biological ages differ more from chronological ages. According to the accuracy test, skeletal and chronological ages do not show a statistical difference in group B.
